# Editorial: Advances and novel technologies in surgical instruments for the treatment of cancer

**DOI:** 10.3389/fonc.2022.1012750

**Published:** 2022-10-04

**Authors:** P.J. Schuler, T.K. Hoffmann, L.A. Kahrs

**Affiliations:** ^1^ Department of Otorhinolaryngology, Head and Neck Surgery, Ulm University Medical Centre, Ulm, Germany; ^2^ Surgical Oncology Ulm, i2SOUL Consortium, Ulm, Germany; ^3^ Department of Mathematical and Computational Sciences, University of TorontoMississauga, Toronto, ON, Canada; ^4^ Institute of Biomedical Engineering, University of Toronto, Toronto, ON, Canada

**Keywords:** cancer, technology, instruments, surgery, imaging

Traditional surgery for patients with malignant diseases is increasingly supported by robotic systems, artificial intelligence, augmented reality and smart instruments. These new techniques help the surgeons to plan and perform the surgical task in a better or faster way. Although in the last years the technical development has produced a long list of excellent opportunities in this field, the majority fails to find their way in into the clinical routine. The reasons often include the questionable benefit for the patients, difficulties in handling the device in a feasible manner or overwhelming costs, which are not covered by the medical insurance. In addition, devices, which are regularly used for the treatment of patients in the clinic, often lack the verification of their benefit by randomized clinical trials.

The goal of the special issue is to bring together technical developers and clinical physicians already in an early stage of development in order to discuss the technical potential and the clinical needs in the treatment of cancer patients. We believe that within every technical development, the patients’ needs and the potential benefit for the treatment of their tumor burden should be in the center of interest. To this end, this special issue offers an open platform for both, technical and clinical contributions, which aim to improve the treatment of oncologic patients by the means of novel technical ideas.

A series of the accepted manuscripts focus on endoscopic techniques in the field of surgery describing procedures in a variety of organs including liver, esophagus, vagina, parotid gland, rectum and prostate. This demonstrates that almost all surgical specialties have adapted to this form of visualization in some form.

A publication reports on the consensus meeting on operating specifications for laparoscopic radical resection of hilar cholangiocarcinoma (HCCA). It lists 16 recommendations for preoperative management of HCCA in a laparoscopic setting. At the same time the experts agree that the laparoscopic approach for HCCA is still in the early phase of development.

A population-based analysis on the surgical therapy for early esophagogastric junction adenocarcinoma has received its data from the worldwide Surveillance, Epidemiology, and End Results (SEER) database including 3,708 patients. The analysis aimed to define the risk for lymph node metastasis comparing the endoscopic versus the open surgical setting. In conclusion, the endoscopic approach is non-inferior to open surgery in terms of risk for lymph node metastasis.

A very similar approach on elderly patients with endometrial cancer also investigated the outcome of laparoscopic surgery. The total study population of 537 patients was retrospectively recruited from seven institutions and divided into groups with laparoscopic or robotic surgery. The study concluded that there was no difference in survival. However robotic surgery was associated with lower blood loss and longer operating times.

The collection of manuscripts is continued by five meta-analysis publications, which compare endoscopic, robotic and other surgical approaches in large patient cohorts. The first analysis included 572 patients with parotid tumors and compared endoscopic/robotic parotidectomy with conventional open parotidectomy (CP) in terms of safety and efficacy. Based on their findings, the authors conclude that endoscopic and robotic parotidectomy should be reserved to those patients with strong cosmetic needs after adequate preoperative evaluation.

The second meta-analysis compared the laparoscopic approach with the robotic approach in 510 patients with intersphincteric resection of low rectal cancer. In contrast to the parotid study, the potential benefits of robotic surgery are considered a safe and feasible alternative for the treatment of low rectal tumors.

Robotic surgery for prostate cancer is considered to be a well-established clinical routine in most developed countries around the world. Different robotic approaches a therefore compared in the following meta-analysis on 3,129 patients with prostate cancer. The authors conclude, that resection of the Retzius space during radical prostatectomy is associated with worse recovery of continence, longer operation time and higher incidence of herniation. Although there was no significant difference in overall survival, this approach should still be considered in lesions of the anterior prostate.

Two meta-analyses reported on the treatment of hepatocellular carcinoma in 803 and 1,158 patients, respectively. The transcatheter arterial chemoembolization in combination with high-intensity focused ultrasound was associated with longer overall survival and should be recommended for patients with advanced hepatocellular carcinoma. In addition, fusion imaging in radiofrequency ablation was reported to have some effects on improving safety and efficacy as compared to standard ultrasound. However, currently fusion imaging should only be recommended for large lesions or those, which are difficult to ablate.

The second group of manuscripts in this special issue deals with novel surgical approaches and their potential benefit for the patients. In these publications, surgeons directly report on their experiences in the treatment of their patients, which make these contributions especially valuable.

The first retrospective interventional cohort study included 366 patients with intraocular tumors and a mean follow-up of 87 months. The authors state, that in their hands partial transscleral sclerouvectomy in combination with microinvasive vitrectomy is safe procedure, which can preserve the usefull vision in these patients and should be discussed as an alternative to enucleation.

Two working groups reported on the surgical therapy of limb tumors. The first retrospective study recommended an extended curettage as a feasible treatment option for giant cell tumor (n=29) of the proximal femur as an alternative to segmental resection. In the second case-series the authors reported on the experience with limb salvage surgery in 56 patients with malignant forearm tumors and gave recommendations for risk evaluation.

Another retrospective study approached the problem of ischemia reperfusion injury in liver transplantation. The authors demonstrated in 226 patients with hepatocellular carcinoma that an ischemic-free surgical procedure can significantly reduce hepatocyte necrosis and apoptosis after liver transplantation.

A small, prospective randomized trial in 30 patients with lung cancer compared to different sequences for the procedural steps during video-assisted thoracoscopic surgery. Another retrospective case-series evaluated the feasibility of endotracheal stent placement in combination with intra-arterial chemoembolization in patient with lung cancer (n=42). The authors demonstrated, that this may be an appropriate approach in this palliative patient collective.

Finally, in a human ex vivo trial (n=12), the authors demonstrated who the quality of fresh urinary tract biopsies can be improved by applying a cryobiopsy technique.

The last three manuscripts focus on the development of instruments and software in order to facilitate surgery.

To this end, a modular MR-compatible robot was developed, which can independently place multiple needles into a tissue for e.g. brachytherapy of the prostate. The whole procedure is guided by MR-imaging.

In a second study, the comparison of cutting qualities between CO_2_ laser and diode-pumped Er-YAG laser was performed on an *ex vivo* animal model. According to the authors, the CO_2_ laser was inferior in terms of cutting efficacy and thermal damage width.

Finally, based on a data set of 195 patients with gallbladder polypoid lesions, the authors have developed a preoperative nomogram of clinical and radiomic features. This nomogram was validated for predicting benign and malignant lesions.

In conclusion, the publications in this special issue present a variety of new surgical approaches and technical developments, which help to better treat our patients with oncologic diseases.

## Author contributions

PJS wrote the manuscript, TKH and LAK reviewed the manuscript. All authors contributed to the article and approved the submitted version.

## Conflict of interest

The authors declare that the research was conducted in the absence of any commercial or financial relationships that could be construed as a potential conflict of interest.

## Publisher’s note

All claims expressed in this article are solely those of the authors and do not necessarily represent those of their affiliated organizations, or those of the publisher, the editors and the reviewers. Any product that may be evaluated in this article, or claim that may be made by its manufacturer, is not guaranteed or endorsed by the publisher.

